# Divergent assembly of soil microbial necromass from microbial and organic fertilizers in *Chimonobambusa hejiangensis* forest

**DOI:** 10.3389/fmicb.2024.1291947

**Published:** 2024-06-10

**Authors:** Xue Cai, Zhijian Long, Yongyang Li, Ying Cao, Boya Wang, Bo Zhao, Peng Ren, Xin Zhao, Yan Huang, Xueqin Lu, Shanglian Hu, Gang Xu

**Affiliations:** School of Life Science and Engineering, Southwest University of Science and Technology, Mianyang, China

**Keywords:** microbial or organic fertilizer, soil microbial residues, soil organic carbon, microbial necromass accumulation coefficient, bamboo forest

## Abstract

**Introduction:**

Variability in microbial residues within soil aggregates are becoming progressively essential to the nutritive and sustainability of soils, and are therefore broadly regarded as an indispensable part of soil organic matter. It is unexplored how the widespread implementation of microbial fertilisers in agricultural production impacts soil organic nutrients, in particular the microbial residue fraction.

**Methods:**

We performed a three-year field experiment to verify the distinct impacts of microbial and organic fertilizers on carbon accumulation in soil microbial leftovers among aggregate fractions.

**Results:**

Microbial residual carbon was shown to decrease insignificantly during the application of microbial fertilizer and to rise marginally afterwards with the utilization of organic fertilizer. However, the combined effects of the two fertilizers had substantial impacts on the accumulation of microbial residual carbon. Changes in the structure of the fungi and bacteria shown in this study have implications for the short-term potential of microbial fertilizer shortages to permanent soil carbon sequestration. Additionally, our findings revealed variations in microbial residue accumulation across the microbial fertilizers, with *Azotobacter chroococcum* fertilizer being preferable to *Bacillus mucilaginosus* fertilizer due to its higher efficiency. In this scenario of nutrient addition, fungal residues may serve as the primary binding component or focal point for the production of new microaggregates, since the quantity of SOC provided by fungal residues increased while that supplied by bacterial residues decreased.

**Discussion:**

Our findings collectively suggested that the mechanisms behind the observed bacterial and fungal MRC (microbial residue carbon) responses to microbial fertilizer or organic fertilizer in bamboo forest soils are likely to be distinct. The application of microbial fertilizers for a limited duration led to a decline soil stable carbon pool, potentially influencing the regulation of soil nutrients in such hilly bamboo forests.

## 1 Introduction

The global soil carbon (C) is considerable large, and even relatively small variations in soil organic carbon (SOC) pools can significantly modify atmospheric C and global climate (Zhu et al., 2020a). Recently, soil microbial carbon pump conceptualization underlined that large soil microbial necromass incorporates into the soil stable C pools more than 50% conceptually (Liang, 2020) and empirically (Liang et al., 2020). A growing number of practical tests on soil microbial remnants contribute to the extent to which SOC stock is rising in agricultural systems (Ding et al., 2015; Li et al., 2020; Poffenbarger et al., 2020; Zhu et al., 2020b). A meta-analysis of publication revealed that fungi accounted for two-thirds of microbial residues, whereas forest soil microbial residues supplied between 34 and 44% of soil organic carbon (Wang B. et al., 2021). Yet, little is known concerning the impact of micro- or organic fertilizer practices on soil microbial residues and their contribution to SOC in bamboo forests.

Soils are generally thought to be a complex of three-dimensional structures consisting of aggregates and pore spaces (Oades and Waters, 1991; Bailey et al., 2013). Aggregates are made up of mineral particles and organic carbon aggregates to provide structural durability against external disturbances and wetting events (Bravo et al., 2018). Aggregates have a hierarchical structure that allows them to form a complex network of particles and cavities in order to achieve periodic connections and complete the flow of nutrients and water through soil organisms (Wilpiszeski et al., 2019). Large marcoraggregates (large than 2 mm in size), small macroaggregates (0.25–2 mm in size), and microaggregates (less than 0.25 mm in dimension) are the typical size classifications for soil aggregates (Jiang et al., 2018; Totsche et al., 2018). On temporal scales, disparities in the inherent features of the components of macroaggregates (which contain more active and labile carbon) and microaggregates (which contain more recalcitrant carbon) generate variations in soil aggregate turnover rates (Christensen, 2001; Totsche et al., 2018; Schweizer et al., 2019). As a functional component and driver of soil ecosystems, soil aggregates play a crucial role in mediating SOC stability and turnover through their physical properties, chemical characteristics, and biological activity in soil microhabitats (Lehmann et al., 2017; Jing et al., 2018; Upton et al., 2019). Soils are primarily a mixture of macro-aggregates, which limit oxygen diffusion and regulate water flow, and micro-aggregates, which bind soil homeostatic organic matter and prevent its removal by erosion (Six et al., 2004; Wilpiszeski et al., 2019). Soil aggregation turnover is thought to be the primary driver of SOC retention, with micro-aggregates stabilizing more soil steady-state carbon fractions from larger aggregates (Six et al., 2004; Yuan et al., 2021). Organic fertilizer utilization has been shown to enhance SOC while also providing a favorable environment for a wider variety of microorganisms (such as fungi and bacteria), with variable results for microbial residue (Bipfubusa et al., 2008; Murugan and Kumar, 2013; Ding et al., 2015; Zhao et al., 2019; Li et al., 2020; Samson et al., 2020). Few studies have focused on the effects of organic fertilizer application on microbial residues in various aggregates (Bipfubusa et al., 2008; Ding et al., 2015). Soil aggregates have the potential to serve as microhabitats for microbial colonization, influencing the spatial distribution of microbial residues inside soil aggregates. It has been shown that fungi tend to exhibit a preference for macroaggregates, whereas bacteria seem to favor microaggregates (Lehmann et al., 2017; Murugan et al., 2019; Yuan et al., 2021). However, there exists a present deficiency in understanding the impact of soil aggregates on the reaction of soil microbial necromass to nutrient inputs, whether they are in the form of organic or microbial fertilizers.

Fertilizers, as indispensable components of modern agriculture and forestry, are being implemented worldwide due to that they can provide essential plant nutrients. However, overapplication of the chemical fertilizer may cause a number of issues, such as soil acidification, nutrient loss, groundwater contamination, heavy metal accumulation, and harmful insects or pathogenic bacteria in the soil (Chen, 2006; Han et al., 2015). Consequently, the combined use of chemical and organic fertilizers such as animal manures, crop residues as well as green manure, and biofertilizers has proved to be highly beneficial on plant quantity and quality with a sustainable effort by changing soil micronutrients (Chen, 2006; Stamenković et al., 2018). For instance, a seven-year field experiment conducted by Chand et al. (2006) on mint and mustard cropping sequence demonstrated a significant effect on soil fertility and crop productivity. Glick et al. (2007) observed that plant-growth-promoting rhizobacteria increased plant growth by synthesizing plant hormones or facilitating the uptake of nutrients from soil in the form of available N and P elements. Lately, numerous researchers have been studying microbial fertilizers as a potential eco-friendly alternative to chemical fertilizers (Stamenković et al., 2018; Bala, 2022). It is beneficial to attempt deploying microbial fertilizers and their coupling with organic fertilizers in field settings to improve crop yields because the majority of the prior researches were conducted under monitored circumstances.

Considering its minimal potential downside, plant growth-promoting microorganisms (PGPMs) have seen growing popularity in recent years in agricultural production and environmental safeguarding. PGPMs boost plant biomass, strengthen root secretion to intensify microbial activities (Zaidi et al., 2015), improve the uptake of essential compounds, and prevent the accumulation of toxic compounds (Yadav et al., 2015). PGPMs acquire much more nitrogen fixation and phosphate solubilization, stimulate plants as biopesticides and rhizomediators through microbial hormones, siderophores, cyanides, and lytic enzymes (Bjelić, 2014), and inhibit pathogens through the production of antibiotics and enzymes (García-Fraile et al., 2015). PGPMs in general can be divided into bacteria and fungi, while bacteria for plant growth promotion can be divided into symbiotic or free-living bacteria (Zaidi et al., 2015). *Azotobacter*, as heterotrophic free-living N_2_-fixing bacteria, is widely investigated and is known to synthesize biologically active growth-promoting substances (Sharma and Bhatnagar, 2005). *Azotobacter chroococcum* is the most commonly occurring species in arable soil (Moraditochaee et al., 2014). Its application can secrete vitamin B complex and different phytohormones that inhibit certain root pathogens but promote root growth and mineral uptake considerably (Babalola, 2010). Moreover, *Bacillus mucilaginosus* is widely used as a microbial fertilizer to release potassium and phosphorus from soil (Liu et al., 2006; Yang et al., 2016). The two microbial fertilizers are commonly used in the lab or greenhouse for testing their effects on plant productivity (Basak and Biswas, 2009; Stamenković et al., 2018). Application of microbial fertilizer on soil organic carbon and aggregate stability has been carried out on a variety of plants (Yilmaz and Sönmez, 2017; Imran and Al Tawaha, 2021; Zhu et al., 2021); however, its application in forestry production is rare, especially in pure bamboo-shoot forests. Additionally, there have been few field studies to investigate the responses of microbial-organic fertilizer to soil microbial leftovers.

Overuse of chemical fertilizer in bamboo forests in recent decades has resulted in a myriad of challenges, such as soil acidification, heavy metal pollution, and quantity and quality of bamboo shoots (Li et al., 2010, 2013). Hence, a plethora of organic fertilizer practices have emerged in various bamboo forests, such as Moso bamboo (Long et al., 2016; Yang et al., 2017; Huang et al., 2021) and *Phyllostachys praecox* (Jiang et al., 2006; Meng et al., 2009; Li et al., 2010). Few researchers focused on the effect of biofertilizer on bamboo plantations and their productions (Hazarika et al., 2006). Currently, the mechanism from the soil surface to plant physiological responses under microbial fertilizer is still open in pure bamboo forests. Moreover, *Chimonobambusa hejiangensis C. D. Chu.*, a special bamboo species only located in a specific area in China, lives in hilly areas ranging from 380 to 1,200 a.s.l., which is regarded as the main economic product for local people by gaining the bamboo shoot (Wang X. et al., 2016). Unfortunately, bamboo shoot yield keeps declining as a result of overuse and untimely replenishment of soil organic matter. Therefore, soil nutrient practices are crucial in this area for increasing soil nutrients and maintaining bamboo shoot productivity. A three-year continuous fertilization research project was employed in this study to explore the impact of microbial fertilizers, organic fertilizers, and their co-application to microbial residues of various aggregates, as well as their contribution to SOC in a subtropical-humid bamboo forest. We put forth two hypotheses. We originally postulated that the uneven availability of nutrients due to the usage of microbial and organic fertilizers would lead to the unequal deposition of fungal and bacterial remnants. We also postulated that fertilizer use would lead to a greater accumulation of bacterial than other microbial leftovers inside soil aggregates. This is because the eutrophic state caused by fertilization would encourage the proliferation of bacteria using an *r*-strategy, which would then result in the consumption of fungal wastes.

## 2 Materials and methods

### 2.1 Experimental site and design

*Chimonobambusa hejiangensis* forest is an indigenous bamboo species that inhabits China’s transition zone between the southern border of the Sichuan basin and the Yunnan-Guizhou plateau (28°18′15″N–28°37′95″N, 105°36′22″E–106°17′48″E). This forest is primarily located in mountainous regions and spans an altitude range of 380 to 1,300 meters. Three sites, founded in March 2017, are in a subtropical monsoon climate zone with a mean annual temperature of 17.9°C and a mean annual precipitation of 1,286 mm. Rainfall occurs primarily from June through October. The climate in this region is humid, with a hot, rainy summer and a cold winter. The Mountain slopes are dominated by the prevalence of *Chimonobambusa hejiangensis,* a species that can attain heights exceeding 4 m and is widely spread, providing a closed canopy. Bamboo litter covers the soil surface approximately 2 cm in depth with various thicknesses and coverages. The major attributes and management history of *Chimonobambusa hejiangensis* forest are presented in Table 1. The soil is a brown soil (USDA, United States Department of Agriculture) derived from feldspar, quartz sandstone, and deluvium. The total carbon of topsoil (20 cm in depth) is 34.11 g kg soil^−1^, total nitrogen is 3.18 g kg soil^−1^, total phosphorus is 0.31 g kg soil^−1^, total potassium is 6.09 g kg soil^−1^, available phosphorus is 1.15 mg kg soil^−1^, and available potassium is 56.67 mg kg soil^−1^. The average pH is 5.94 ± 0.26, and the soil bulk density is 0.89 g cm^−3^. In this study, a randomized block design was employed at each site with a total of nine treatments, as shown below: The experimental treatments included: (1) the absence of any fertilizers (CK); (2) the application of only microbial fertilizer (*Bacillus mucilaginosus*) at a rate of 15.20 kg/ha (BMF); (3) the application of only microbial fertilizer (*Azotobacter chroococcum*) at a rate of 15.20 kg/ha (ACF); (4) the application of a organic fertilizer at a rate of 1,520 kg/ha (BOF); (5) the application of special organic fertilizer particularly designed for bamboo shoots at a rate of 1,520 kg/ha (BSF); (6) the application of organic fertilizer at a rate of 1,520 kg/ha, combined with *Bacillus mucilaginosus* at a rate of 15.20 kg/ha (BOF + BMF); (7) the application of organic fertilizer at a rate of 1,520 kg/ha, paired with *Azotobacter chroococcum* at a rate of 15.20 kg/ha (BOF + ACF); (8) the application of special organic fertilizer specifically designed for bamboo shoots at a rate of 1,520 kg/ha, mixed with *Bacillus mucilaginosus* at a rate of 15.20 kg/ha (BSF + BMF); (9) the application of special organic fertilizer specifically designed for bamboo shoots at a rate of 1,520 kg/ha, utilized with *Azotobacter chroococcum* at a rate of 15.20 kg/ha (BSF + ACF). Specifically, BOF contains nutrient elements with an amount of N 0.2%, P 1.1%, K 5.5%, respectively, and BSF includes nutrient elements with N 1.4%, P 4.5%, K 1.7%, respectively. BMF (a microbial agent with 10 billion CFU/g) decomposing potassium, silicon, and phosphorus from soil minerals and ACF (a microbial agent with 10 billion CFU/g) as nitrogen-fixing biofertilizers were purchased from Huanwei Biology Corporation, China. The determination of the application rate of organic fertilizers was based on reference the customary quantity of fertilizers applied by farmers within the local agricultural community. On the contrary, the conventional practice of applying microbial fertilizers relies on the prescribed dosage suggested by the respective producers of these fertilizers. It is recommended to use microbial fertilizers at a proportionate rate of 1% in relation to the application rate of organic fertilizers. Microbial fertilizer, including BMF and ACF, is applied by spreading 15.20 kg per hectare, namely 1.520 g/m^2^. There were three blocks, and the distance between the blocks ranged from 50 to 100 m. There are twenty-seven plots in each block, and the area of each plot is 20 × 20 m with three replicates. The fertilizer was sprayed all at once every March.

**Table 1:** General attributes of *Chimonobambusa hejiangensis* forest.

	*Chimonobambusa hejiangensis* forest
Site description	Pure *Chimonobambusa hejiangensis* forest established after slash and burn of *Cunninghamia lanceolata* plantation forest, which was introduced in 1959. *Chimonobambusa hejiangensis* with a height of 25–40 cm were cultivated with an initial density of 2,500–3,500 stem hm^−2^ according to site condition. We only applied fertilizer at rates of 100 kg N hm^−2^ and 60 kg P_2_O_5_ hm^−2^ during the first year. After that, this area was remained without any fertilizer. Annual bamboo-shoot harvesting carried out after three-year cultivation of *Chimonobambusa hejiangensis*
Dominant tree species	*Chimonobambusa hejiangensis*
Slope (°)	39–68
Mean stand age (years)	56
Mean tree height (m)	3.2
Diameter at basal stem (cm)	2.059
Tree density (stem hm^−2^)	28,125
Total annual litter (t hm^−2^)	1.33
Litter C/N	26.75
Total forest floor biomass (t hm^−2^)	3.48

### 2.2 Soil sampling

Soil samples were taken in October 2018, 2019, and 2020, correspondingly. Five complete soil cores at a depth of 0 to 20 cm were randomly selected and thoroughly mixed to create composite samples for each plot. The freshly harvested soils were sieved through a 4-millimeter mesh to remove debris including roots, boulders, and plants. Cooling boxes were utilized to transport soil samples to the lab for additional investigation. Sub-mixed soil samples were stored at 4°C for less than a week for microbial characteristics and enzymatic activities and at −80°C for soil phospholipid fatty acid (PLFA) analysis until processing. The soil samples for Next-Generation Sequencing were collected in October 2020, with 9 biological replicates for each treatment. The remaining soil samples were air-dried at indoor temperature and ground for the analysis of physicochemical and microbial residue attributes. The air-dried soil was fractioned into three particle-size levels through a nest of three sieves (4, 2, and 0.25 mm): large-aggregate soil (2–4 mm), macro-aggregate soil (0.25–2 mm), and micro-aggregate (<0.25 mm) by an electric vibrating sifter (model 8411, Shanghai Leiyun Test Equipment Manufacture Co., China).

### 2.3 Soil physicochemical properties

A pH meter (FiveEasy Plus^™^ FE28, Mettler Toledo) was utilized to evaluate soil pH level using a 1:5 (w/v) soil-water combination. Shimadzu TC (Shimadzu Company, Kyoto, Japan) was applied to determine SOC, while a Carlo Erba elemental analyzer was used to evaluate soil total nitrogen (Carlo-Erba, Milan, Italy). The measurement of total phosphorus (TP) in soil samples was conducted using a continuous-flow chemical analyzer (AA3, Seal Analytical, Norderstedt, Germany). This included submitting the soil samples to acid digestion using sulfuric acid (H_2_SO_4_) and perchloric acid (HClO_4_). The TP levels were then detected using a UV spectrophotometer at a wavelength of 680 nm.

### 2.4 Soil microbial attributes and enzymatic activities

The characteristics of soil PLFA fractions were evaluated according to Frostegård et al. (1991) and Hackl et al. (2005), with slight modifications. Briefly, 10 g of freeze-dried soil samples were extracted using a buffer composed of chloroform-methanol-phosphate (1:2:0.8, v/v/v). In a 0.5 g silica acid column, the extracted lipids were sorted into neutral lipids, glycolipids, and phospholipids. The extracted fatty acid methyl esters were trans-esterified to fatty acid methyl esters by mild alkaline methanolysis and then analyzed via GC-MS (Hewlett-Packard 6890 N-5973 N). Peaks were identified with bacterial fatty acid standards and Sherlock peak identification software (MIDI, Inc., Newark, DE). Nonadeconoic methyl ester (19:0) was used as an internal standard peak for comparisons of peak areas from the samples (Smithwick et al., 2005). The PLFAs i14:0, i15:0, α15:0, i16:1, α16:0, i16:0, i17:0, α17:0, and i17:0 were used to represent the gram-positive bacteria. The monounsaturated and cyclopropyl saturated peaks representing 16:1 ω7c, 16:1 ω9c, cy17:0, cy19:0, 2OH 16:1, 3OH 11:0, and 2OH 18:1 were used as indicators of gram-negative bacteria (Xu et al., 2015). The total size of soil bacteria was calculated by adding Gram-positive and Gram-negative bacterial markers. Furthermore, the peaks 18:3 ω6c, 18:1 ω9c, 18:1 ω5c, and 18:1 ω7c were used as indicators of non-specific fungi. Arbuscular mycorrhizal and ectomycorrhizal fungi were represented by the typical peaks of 16:1 ω5c and 18:2, ω6, 9c, respectively. The total fungal biomass was obtained by adding unspecific fungi, arbuscular mycorrhizal fungi, and ectomycorrhizal fungi (Kaur et al., 2005).

Two extracellular soil enzymes, β-1, 4-glucosidase (BG) and β-1, 4-N-acetylglucosaminidase (NAG), were determined according to the protocol of Paz-Ferreiro et al. (2012). P-nitrophenyl-β-d-glucopyranoside and p-nitrophenyl-N-acetyl-β-d-glucosaminide (Sigma) were specific substrates for the determination of BG and NAG, respectively. Using a microplate photometer (Multiskan^™^ FC, Thermo Fisher Scientific, Waltham, MA, United States), the absorbance of each sample was determined at 405 nm, and enzyme activities were expressed as μmol g^−1^ dry soil hr^−1^. Soil acid phosphatase (AP) was determined with para-nitrophenyl phosphate (p-NPP) as substrate, as described by Yuan et al. (2021). A spectrophotometer was used to determine the concentration of para-nitrophenol at 400 nm. AP activity is expressed as μmol g^−1^ soil h^−1^.

### 2.5 DNA extraction, library construction, and metagenomic sequencing

To get the total genomic DNA, 1 g of each soil was ground twice with nitrogen gas and then processed following the instructions provided by the manufacturer using the Mag-Bind^®^ Soil DNA Kit (Omega Bio-tek, Norcross, GA, United States). The concentration and purity of the extracted DNA were assessed using the TBS-380 and NanoDrop2000 instruments, respectively. The quality of the DNA extract was assessed using a 1% agarose gel. The polymerase chain reaction (PCR) test was solely employed to amplify the V3–V4 sections of the 16S rRNA gene in soil bacteria using specific primers. The primers utilized for amplification were F515 (5′-GTGC CAGCMGCCGCGGTAA-3′) and R907 (5′-CCGTCAA TTCMTTTRAGTTT-3′) as described by Chen et al. (2021). The ITS region, which serves as the global DNA barcode identifier for soil fungi, was amplified using the primers ITS1F (5′-GGAAGTAAAAGTCGTAACAAGG-3′) and ITS2R (5′-GCTGC GTTCTTCATCGATGC-3′). After purifying the amplicons, they were combined in equal amounts and subjected to paired-end sequencing (2 × 300 bp) using the Illumina MiSeq platform (Guhe Information Technology Co. Ltd.). A paired-end library was created using the NEXTFLEX Rapid DNA-Seq kit from Bioo Scientific, located in Austin, TX, United States. Adapters containing all the sequencing primer hybridization sites were joined to the blunt ends of the fragments by ligation. The Illumina NovaSeq platform, specifically the NovaSeq 6000 S4 Reagent Kit v1.5 (300 cycles), was used for paired-end sequencing. The sequencing was conducted at Majorbio Bio-Pharm Technology Co., Ltd. in Shanghai, China, following the manufacturer’s instructions provided on www.illumina.com. The sequence data pertaining to this experiment have been submitted to the NCBI Short Read Archive database. The data were analyzed using the free online platform provided by Majorbio Cloud Platform.^1^ In summary, the paired-end Illumina reads underwent adaptor trimming and the removal of low-quality reads (reads with a length less than 50 bp, a quality value lower than 20, or containing N bases) using fastp (https://github.com/OpenGene/fastp, version 0.20.0). Sequences that had a similarity of 97% or higher were grouped together into operational taxonomic units (OTUs) using Vsearch (v2.3.4). To minimize over splitting and sequencing mistakes, OTUs that were assigned to chloroplast or mitochondria, or had less than four reads in all samples, were filtered out.

A total number of 410,982 valid sequencing reads were acquired for bacteria, with 369,812 reads resulting from clustering the genomes. After filtering, 5,132 OTUs were identified based on the sequences and used for downstream analysis. A total of 812,759 and 192,518 valid sequencing reads were recovered for fungus. The soil samples produced a total of 182,427 reads from their sequencing. For the downstream analysis, a grand total of 1,179 OTUs were utilized for the soil samples by removing sequences of chloroplast. In order to standardize the OUT abundance data, the sample with the least sequences was used as a reference. The observed species indices, Chao1, Shannon, and Simpson, as well as the alpha diversity index, were computed with QIIME (v1.8.0) to assess the complexity of species diversity in a given sample.

### 2.6 Soil amino sugars

Soil amino sugars were extracted according to the protocol described by Zhang and Amelung (1996). Briefly, 1 g of soil was hydrolyzed with 10 mL of 6 M HCI at 105°C for 6 h. After hydrolysis, the samples were mixed well and filtered. The pH was adjusted to 6.6–6.8 by re-suspending the samples with deionized water. To remove hydrochloric acid, the supernatant was blown dry with nitrogen gas. The amino sugar derivatives were re-dissolved in 200 μL of ethyl acetate-hexane (1:1) for final analysis after removing dichloromethane with a nitrogen stream. Finally, the amino sugar derivatives were separated on an Agilent 7890 B gas chromatograph (Agilent Technologies, California, United States). The microbially produced soil amino sugar fraction in the present study consisted of glucosamine (GluN), galactosamine (GlaN), muramate (MurA), and mannosamine (ManN). The concentration of soil amino sugars was expressed as mg kg^−1^ dry soil. Microbial residual carbon (MRC) equals fungal residual carbon (FRC) plus bacterial residual carbon (BRC). The referenced calculation Eqs. 1, 2 are utilized to determine FRC and BRC, respectively (Ni et al., 2020). These equations are expressed in milligrams per kilogram of desiccated soil.


(1) $$\begin{array}{l} FRC\;\left(\mathrm{mg}\;{\mathrm{kg}}^{-1}\right)\\ =\left(\mathrm{GluN}\;\left(\mathrm{mg}\;{\mathrm{kg}}^{-1}\right)/179.2-2\times \mathrm{MurA}\;\left(\mathrm{mg}\;{\mathrm{kg}}^{-1}\right)/251.2\right)\\ \times \;179.2\times 9 \end{array}$$

(2) $$BRC\;\left(\mathrm{mg}\;{\mathrm{kg}}^{-1}\right)=\mathrm{MurA}\;\left(\mathrm{mg}\;{\mathrm{kg}}^{-1}\right)\times 45$$

The proportion of microbial residue carbon in SOC represents the contribution of microbial residue to SOC sequestration. The ratio of the amount of carbon in microbial necromass to the amount of carbon in living biomass is known as the microbial necromass accumulation coefficient (NAC-C). The fumigation-extraction technique, as described by Xu et al. (2015), is employed to estimate the living biomass existing. This shows how much necromass was made per unit of microbial biomass carbon. NAC was calculated by Eq. 3 according to the method described in Wang C. et al. (2021).


(3) NAC−C=Necromasscarbon/Livingbiomasscarbon

### 2.7 Data analysis

The data were subjected to the Shapiro–Wilk test for normality and the Levene test for homogeneity of variance. A one-way ANOVA was conducted using Origin software (OriginLab, OriginPro 2022, United States) to investigate the effects of organic and microbial fertilizer on soil physicochemical, microbial, amino sugar, and microbial OUTs properties. Fisher’s LSD test was performed for the evaluation of differences between fertilizer additions with a probability level of 0.05 when ANOVA showed significant effects. Partial least squares path modeling (PLS-PM) was applied to further identify the possible pathways by which measured variables mediate the response of soil microbial residues and their contribution to the effects of fertilizer additions, using the “plspm” package of RStudio (2023.03.0). Blocks of reflective indicators were defined by latent variables in PLS-PM. Nine blocks of reflective indicators were defined as “Aggregate fraction,” “Nutrients,” “Microbial biomass,” “Microbial activity,” “Amino sugars,” “FRC,” “BRC,” “FRC/SOC,” and “BRC/SOC.” Specifically, “aggregate fraction” was regarded as a factor, and distinct numbers were applied to indicate large-aggregate soil, macro-aggregate soil, and micro-aggregate soil. “Nutrients” were represented by the concentrations of SOC, total N, and total P. “Microbial biomass” was indicated by latent variables of MBC, PLFAs, fungal PLFAs, bacterial PLFAs, and microbial OUTs content. “Microbial activity” was indicated by three soil extracellular enzymes (BG, NAG, and AP). “Amino sugars” were indicated by four latent variables: GluN, GlaN, MurA, and ManN. The goodness-of-fit index (> 0.7) was achieved to verify the model’s validity.

## 3 Results

### 3.1 Soil basic attributes and microbial properties

Generally, fertilization in our study enhanced soil nutrients (SOC) to variable degrees at distinct soil particle-size scales, with the co-application of microbial and organic fertilizers providing a greater increase in soil nutrients than microbial or organic fertilizers alone (Table 2). BSF + ACF considerably raised the concentrations of TN and TP in macro- and micro-aggregate soils. Nonetheless, neither soil C/N nor pH values were substantially altered by applying organic or microbial fertilizer (Table 2). The results showed that BSF + ACF substantially enhanced soil MBC, PLFAs, fungal PLFAs, and bacterial PLFAs in various soil aggregate fractions. However, there was not a statistically significant distinction in the fungal/bacterial ratio (Table 2). Moreover, the quantity of microorganisms generated by ACF was larger than that of BMF, although this variability was not statistically significant (Table 2).

**Table 2:** Basic properties of soil samples (0–20 cm) under different fertilizer treatments in *Chimonobambusa hejiangensis* forest.

	SOC (g kg^−1^)	TN (g kg^−1^)	TP (g kg^−1^)	C/N	pH	MBC (mg kg^−1^)	PLFAs (nmol g^−1^)	Fungal (nmol g^−1^)	Bacterial (nmol g^−1^)	F:B
**Large-aggregate soils**										
CK	26.78 ± 1.74d	2.34 ± 0.21de	0.24 ± 0.03c	13.66 ± 0.18a	5.72 ± 0.62a	422.61 ± 29.59c	25.97 ± 3.56bc	4.53 ± 0.38c	10.28 ± 0.94c	0.44 ± 0.03ab
BMF	27.26 ± 3.06d	2.35 ± 0.15e	0.26 ± 0.03bc	12.54 ± 0.66a	6.13 ± 0.88a	470.36 ± 45.59c	23.65 ± 1.05c	4.55 ± 0.24c	12.83 ± 1.27abc	0.38 ± 0.05b
ACF	26.80 ± 4.00d	2.09 ± 0.28de	0.23 ± 0.03c	13.07 ± 1.73a	5.86 ± 0.71a	480.44 ± 28.27c	30.63 ± 2.92bc	5.01 ± 0.41bc	14.72 ± 0.76ab	0.38 ± 0.03b
BOF	26.01 ± 2.29d	2.54 ± 0.19de	0.24 ± 0.03bc	12.97 ± 0.91a	6.29 ± 0.45a	428.17 ± 47.87c	28.69 ± 3.36bc	4.65 ± 0.50c	13.43 ± 1.55ab	0.43 ± 0.03ab
BSF	32.92 ± 3.10 cd	2.97 ± 0.32bcd	0.25 ± 0.02bc	12.79 ± 0.80a	6.26 ± 0.08a	510.79 ± 63.80c	32.42 ± 2.82ab	4.38 ± 0.58c	11.93 ± 1.77bc	0.40 ± 0.04ab
BOF + BMF	32.84 ± 4.07cd	2.86 ± 0.37cd	0.24 ± 0.04bc	12.61 ± 1.61a	6.65 ± 0.67a	484.94 ± 69.42c	33.64 ± 4.11ab	5.03 ± 0.49bc	12.63 ± 1.37abc	0.44 ± 0.05ab
BOF + ACF	40.87 ± 5.05c	3.42 ± 0.35bc	0.31 ± 0.04ab	13.67 ± 0.73a	5.86 ± 0.77a	662.76 ± 53.37b	39.56 ± 3.74a	6.03 ± 0.69ab	13.88 ± 1.56ab	0.47 ± 0.06a
BSF + BMF	49.73 ± 4.16b	3.72 ± 0.45b	0.26 ± 0.02bc	12.78 ± 0.86a	6.34 ± 0.76a	649.27 ± 63.14b	32.74 ± 3.84ab	5.04 ± 0.71bc	13.54 ± 1.55ab	0.42 ± 0.05ab
BSF + ACF	58.66 ± 6.92a	5.11 ± 0.69a	0.36 ± 0.04a	13.26 ± 1.88a	6.53 ± 0.82a	848.11 ± 87.55a	40.36 ± 6.96a	6.76 ± 0.61a	15.23 ± 1.67a	0.45 ± 0.05ab
**Macro-aggregate soils**										
CK	29.02 ± 4.29e	2.26 ± 0.29d	0.24 ± 0.01b	14.59 ± 1.55a	6.48 ± 0.84a	508.55 ± 64.03c	29.82 ± 2.52c	5.13 ± 0.51c	10.96 ± 1.20c	0.53 ± 0.05a
BMF	29.49 ± 3.51e	2.52 ± 0.22d	0.29 ± 0.04b	13.67 ± 1.41a	6.61 ± 0.57a	501.92 ± 48.21c	29.21 ± 4.12c	5.25 ± 0.75c	14.83 ± 1.71ab	0.42 ± 0.06ab
ACF	29.73 ± 2.64e	2.52 ± 0.27d	0.24 ± 0.03b	14.41 ± 1.74a	6.62 ± 0.83a	497.22 ± 33.56c	34.54 ± 4.34c	5.80 ± 0.53bc	15.15 ± 1.90ab	0.41 ± 0.06b
BOF	29.60 ± 3.32e	2.53 ± 0.37d	0.27 ± 0.02b	13.67 ± 2.11a	6.56 ± 0.70a	500.06 ± 48.52c	34.29 ± 2.85cb	5.06 ± 0.58c	13.86 ± 1.70bc	0.45 ± 0.06ab
BSF	39.68 ± 3.33cd	2.95 ± 0.33cd	0.25 ± 0.01b	14.89 ± 2.25a	6.80 ± 0.88a	587.17 ± 68.84bc	34.48 ± 3.50c	4.94 ± 0.62c	12.35 ± 0.66bc	0.43 ± 0.06ab
BOF + BMF	33.28 ± 4.28de	3.03 ± 0.22cd	0.25 ± 0.03b	13.12 ± 1.44a	7.31 ± 0.69a	594.62 ± 60.10bc	37.61 ± 2.74abc	6.05 ± 0.61abc	12.90 ± 1.70bc	0.50 ± 0.07ab
BOF + ACF	44.10 ± 4.46bc	3.41 ± 0.44bc	0.3 ± 0.03b	14.82 ± 1.85a	7.08 ± 0.78a	685.66 ± 66.71b	40.32 ± 2.60ab	6.80 ± 0.66ab	14.72 ± 0.72ab	0.50 ± 0.07ab
BSF + BMF	53.01 ± 5.13ab	3.87 ± 0.51b	0.27 ± 0.02b	15.60 ± 2.06a	7.59 ± 0.55a	714.71 ± 96.15b	36.59 ± 3.05bc	5.72 ± 0.62bc	14.59 ± 2.21ab	0.45 ± 0.06ab
BSF + ACF	63.36 ± 8.91a	5.31 ± 0.71a	0.37 ± 0.05a	14.93 ± 2.20a	7.33 ± 0.74a	980.64 ± 97.48a	46.42 ± 5.64a	7.23 ± 0.55a	17.30 ± 1.82a	0.48 ± 0.03ab
**Micro-aggregate soils**										
CK	31.00 ± 3.60d	2.50 ± 0.29d	0.29 ± 0.02bc	14.34 ± 1.68a	6.60 ± 0.53a	541.65 ± 66.68cd	28.54 ± 3.14d	5.21 ± 0.52b	10.37 ± 0.88c	0.57 ± 0.07a
BMF	33.36 ± 1.47d	2.72 ± 0.38d	0.31 ± 0.03bc	15.38 ± 1.35a	7.06 ± 0.75a	529.95 ± 72.48cd	31.00 ± 3.42cd	5.17 ± 0.53b	15.73 ± 1.43ab	0.43 ± 0.06bc
ACF	29.78 ± 2.52d	2.68 ± 0.27d	0.26 ± 0.04c	13.77 ± 1.73a	6.91 ± 0.70a	589.84 ± 29.12cd	33.04 ± 4.05cd	5.80 ± 0.39ab	15.90 ± 2.34ab	0.41 ± 0.03bc
BOF	32.52 ± 4.07d	2.9 ± 0.30cd	0.27 ± 0.04c	13.96 ± 2.00a	6.45 ± 0.86a	493.66 ± 31.95d	34.86 ± 3.74cd	5.20 ± 0.65b	14.42 ± 1.57ab	0.47 ± 0.06abc
BSF	39.24 ± 4.09cd	3.32 ± 0.29cd	0.30 ± 0.04bc	14.87 ± 1.53a	7.44 ± 1.03a	547.39 ± 69.15cd	34.33 ± 2.06cd	5.10 ± 0.69b	13.98 ± 1.68ab	0.41 ± 0.03c
BOF + BMF	33.54 ± 4.54d	2.85 ± 0.27d	0.27 ± 0.02c	14.04 ± 2.04a	7.37 ± 0.83a	537.84 ± 40.49cd	37.74 ± 3.14bc	6.08 ± 0.97ab	13.37 ± 1.47bc	0.56 ± 0.04a
BOF + ACF	46.86 ± 4.99bc	3.76 ± 0.42bc	0.35 ± 0.06ab	14.90 ± 1.97a	7.13 ± 0.74a	674.70 ± 81.79bc	43.35 ± 4.69ab	7.15 ± 0.76a	16.04 ± 1.45ab	0.54 ± 0.06a
BSF + BMF	53.19 ± 6.51b	4.23 ± 0.43b	0.31 ± 0.04bc	15.47 ± 1.28a	7.33 ± 0.77a	807.47 ± 93.65ab	37.49 ± 2.36bc	5.48 ± 0.69b	15.85 ± 1.51ab	0.42 ± 0.03bc
BSF + ACF	66.71 ± 9.04a	5.47 ± 0.79a	0.42 ± 0.02a	15.22 ± 1.77a	7.43 ± 0.75a	948.28 ± 112.74a	47.35 ± 3.35a	7.02 ± 0.77a	17.44 ± 2.35a	0.52 ± 0.05ab

The values not sharing the same letter are significantly different (*p* < 0.05) according to Fisher’s LSD test. The values are means ± SD, *n* = 9. CK means no fertilizers; BMF means *Bacillus mucilaginosus* fertilizer; ACF means *Azotobacter chroococcum* fertilizer; BOF means organic fertilizer; BSF means special organic fertilizer for bamboo shoots; BOF + BMF means organic fertilizer plus *Bacillus mucilaginosus*; BOF + ACF means organic fertilizer plus *Azotobacter chroococcum*; BSF + BMF means special organic fertilizer for bamboo shoots plus *Bacillus mucilaginosus*; BSF + ACF means special organic fertilizer for bamboo shoots plus *Azotobacter chroococcum*. SOC means soil organic carbon; TN means total nitrogen; TP means total phosphorus; MBC means microbial biomass carbon; PLFAs means phospholipid fatty acid biomarker; Fungal means total fungi PLFAs; bacterial means total bacterial PLFAs. Large-aggregate soil (2–4 mm), macro-aggregate soil (0.25–2 mm), and micro-aggregate (<0.25 mm).

**Figure 1:**
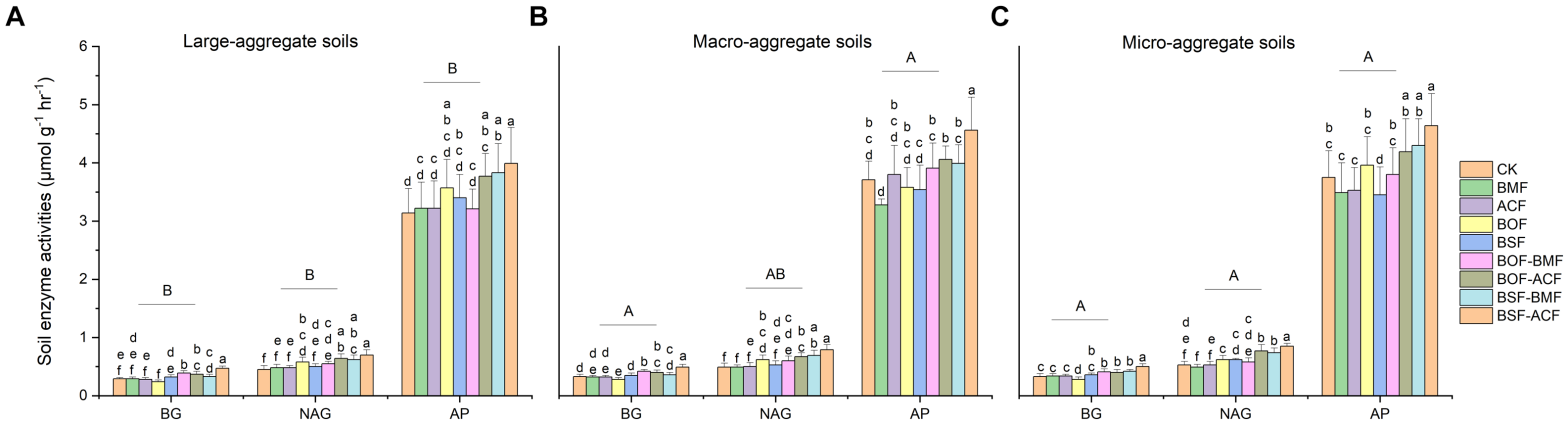
Soil extracellular enzymatic activities under the micro-organic fertilizers in *Chimonobambusa hejiangensis* forest. The values not sharing the same lowercase letters in the bars are significantly different (*p* < 0.05) according to Fisher’s LSD test. The values are means ± SD, *n* = 9. Different uppercase letters indicate significant differences among soil aggregate fractions regardless of fertilizer type (Fisher’s LSD test, *p* < 0.05). CK means no fertilizers. BMF means *Bacillus mucilaginosus* microbial fertilizer. ACF means *Azotobacter chroococcum* microbial fertilizer. BOF means organic fertilizer. BSF means special organic fertilizer for bamboo shoots. BOF + BMF means organic fertilizer plus *Bacillus mucilaginosus*. BOF + ACF means organic fertilizer plus *Azotobacter chroococcum*. BSF + BMF means special organic fertilizer for bamboo shoots plus *Bacillus mucilaginosus*. BSF + ACF means special organic fertilizer for bamboo shoots plus *Azotobacter chroococcum*. BG means β-1, 4-Glucosidase; NAG means β-1, 4-N-acetyl-glucosaminidase; AP means acid phosphatase. Large-aggregate soil (2–4 mm), macro-aggregate soil (0.25–2 mm), and micro-aggregate (<0.25 mm).

In this work, we discovered that BSF + ACF dramatically boosted the activity of three soil enzymes (BG, NAG, and AP) in soils of varying soil particle sizes (Figure 1). The combination of organic and microbial fertilizers considerably boosted BG activity across different aggregate fractions, whereas BG enzyme activity in macro- and micro- fractions was much greater than in large-aggregate soil without considering the fertilization types (Figure 1). NAG activity was increased in BOF + ACF, BSF + BMF, and BSF + ACF in all aggregate fractions (Figure 1). AP activity had differentially increased in large-aggregate soils under all fertilizations (Figure 1A), but showed a decreasing trend in macro- and micro-fractions under BMF and BSF (Figures 1B,C). Irrespective of the type of fertilization, the BG and AP activities were significantly higher in micro and macro soils than in large-aggregate soils, whereas the NAG activity was dramatically higher only in micro-aggregate soils than in large-aggregate soils (Figure 1).

### 3.2 Soil microbial residue attributes

**Figure 2:**
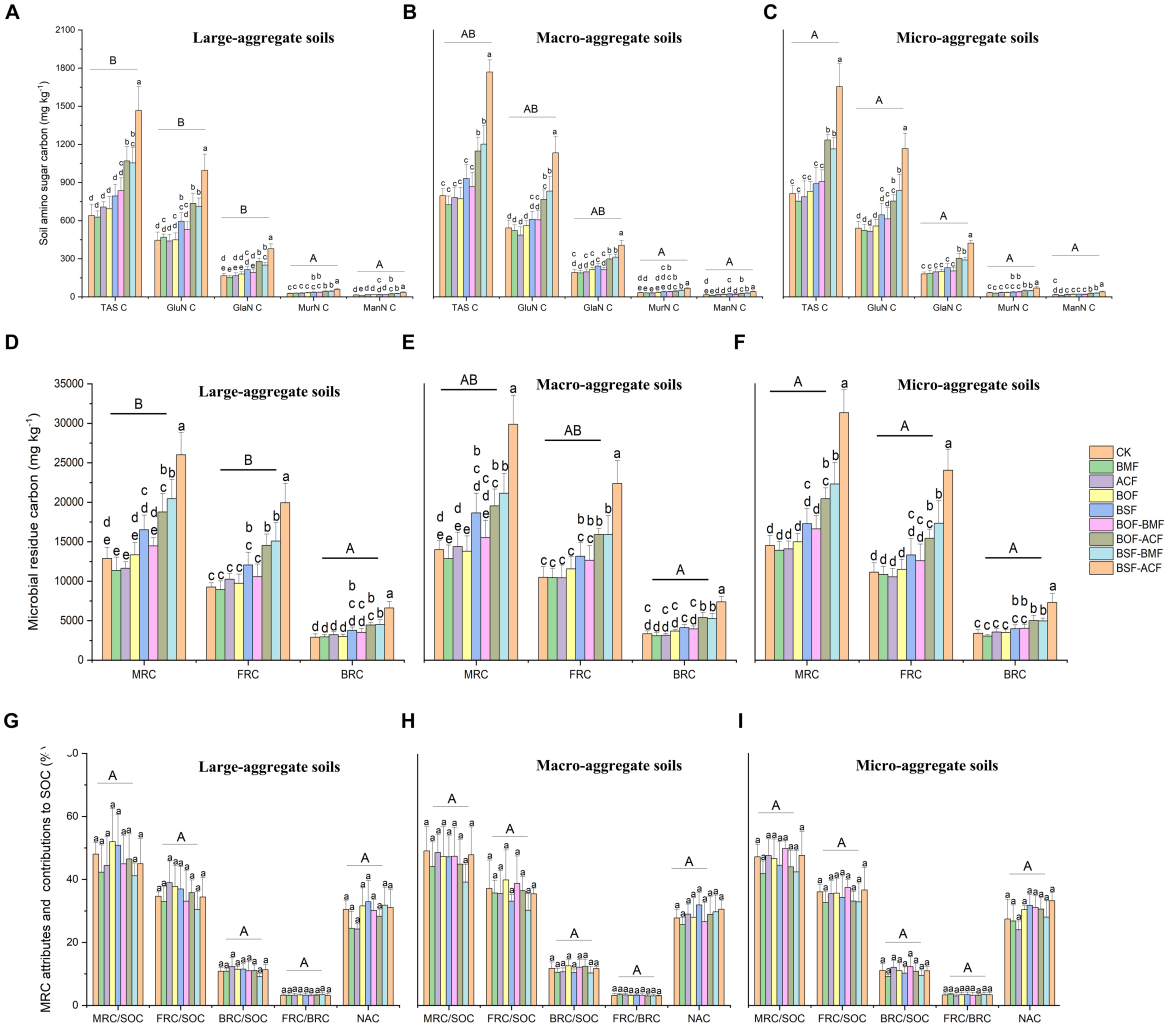
Soil amino sugar attributes, microbial residue carbon concentration, and related attributes under the micro-organic fertilizers in *Chimonobambusa hejiangensis* forest. The values not sharing the same lowercase letters in the bars are significantly different (*p* < 0.05) according to Fisher’s LSD test. The values are means ± SD, *n* = 9. Different uppercase letters indicate significant differences among soil aggregate fractions regardless of fertilizer type (Fisher’s LSD test, *p* < 0.05). CK means no fertilizers. BMF means *Bacillus mucilaginosus* microbial fertilizer. ACF means *Azotobacter chroococcum* microbial fertilizer. BOF means organic fertilizer. BSF means special organic fertilizer for bamboo shoots. BOF + BMF means organic fertilizer plus *Bacillus mucilaginosus*. BOF + ACF means organic fertilizer plus *Azotobacter chroococcum*. BSF + BMF means special organic fertilizer for bamboo shoots plus *Bacillus mucilaginosus*. BSF + ACF means special organic fertilizer for bamboo shoots plus *Azotobacter chroococcum*. Large-aggregate soil (2–4 mm), macro-aggregate soil (0.25–2 mm), and micro-aggregate (<0.25 mm). GluN, glucosamine; GlaN, galactosamine; MurA, muramate; ManN, mannosamine; MRC, microbial residual carbon; FRC, fungal residual carbon; BRC, bacterial residual carbon.

As a whole, soil amino sugar carbon (TAS C, GluN C, GalN C, MurN C, and ManN C) was substantially increased under BSF + BMF and BSF + ACF to different magnitudes, with the highest values in BSF + ACF among soil aggregate fractions (Figures 2A–C). The soil amino sugar carbon concentration was greater under BOF + ACF, BSF + BMF, and BSF + ACF than that under microbial or organic fertilizer alone or the controls (Figures 2A–C). Organic plus microbial fertilizers had a higher potential to enlarge soil microbial necromass carbon accumulation when compared with individual fertilizations and the controls. Microbial fertilizers, in particular, had no significant impact on soil amino sugar carbon concentration in various soil aggregates (Figures 2A–C). Regardless of fertilizer type, we observed a substantial rise in TAS, GluN, and GalN in micro-aggregate soils compared to large aggregate soils, while MurN and ManN were not significantly different (Figures 2D–F).

On a whole, BOF + ACF, BSF + BMF, and BSF + ACF remarkable boosted microbial residue carbon, including FRC and BRC concentrations across different aggregate fractions (Figures 2D–F). BSF alone was more efficient than BMF, ACF, BOF, and BOF + BMF in accumulating soil microbial residual carbon but less effective than BOF + BMF, BSF + BMF, and BSF + ACF among differing particle-size aggregates (Figures 2D–F). The application of microbial fertilization (BMF and ACF) alone did not lead to a significant increase in microbial residues compared to the controls (Figures 2D–F). This is probably since a bio-fertilizer (Dębska et al., 2016) was added, which improved the stability of the stable fractions of organic matter rather than the original microbial residues first. Independent of fertilization type, there were considerable differences in soil MRC and FRC in large-aggregate soils and micro-aggregate soils, with micro-aggregate soils being more conducive to their accumulation (Figures 2D,F). Moreover, regardless of fertilizer type, soil BRC did not reveal significant changes in various aggregate fractions, showing that soil aggregate size variances had little effect on the accumulation of bacterial residues in this bamboo forest. Overall, fertilization strategies reduced the contribution of microbial residual carbon to SOC (Figures 2G–I). The contribution of fungal and bacterial residues to SOC increased slightly in large aggregates but decreased in macro- and micro- aggregates (Figures 2G–I). Furthermore, the accumulation coefficient of microbial residues slightly decreased in large aggregates while increased slightly in macro- and micro-aggregates (Figures 2G–I). The MRC/SOC, FRC/BRC, and NAC did not differ significantly among particle-size aggregates, irrespective of the fertilizer types (Figures 2G–I).

### 3.3 Soil microbial compositional traits

**Figure 3:**
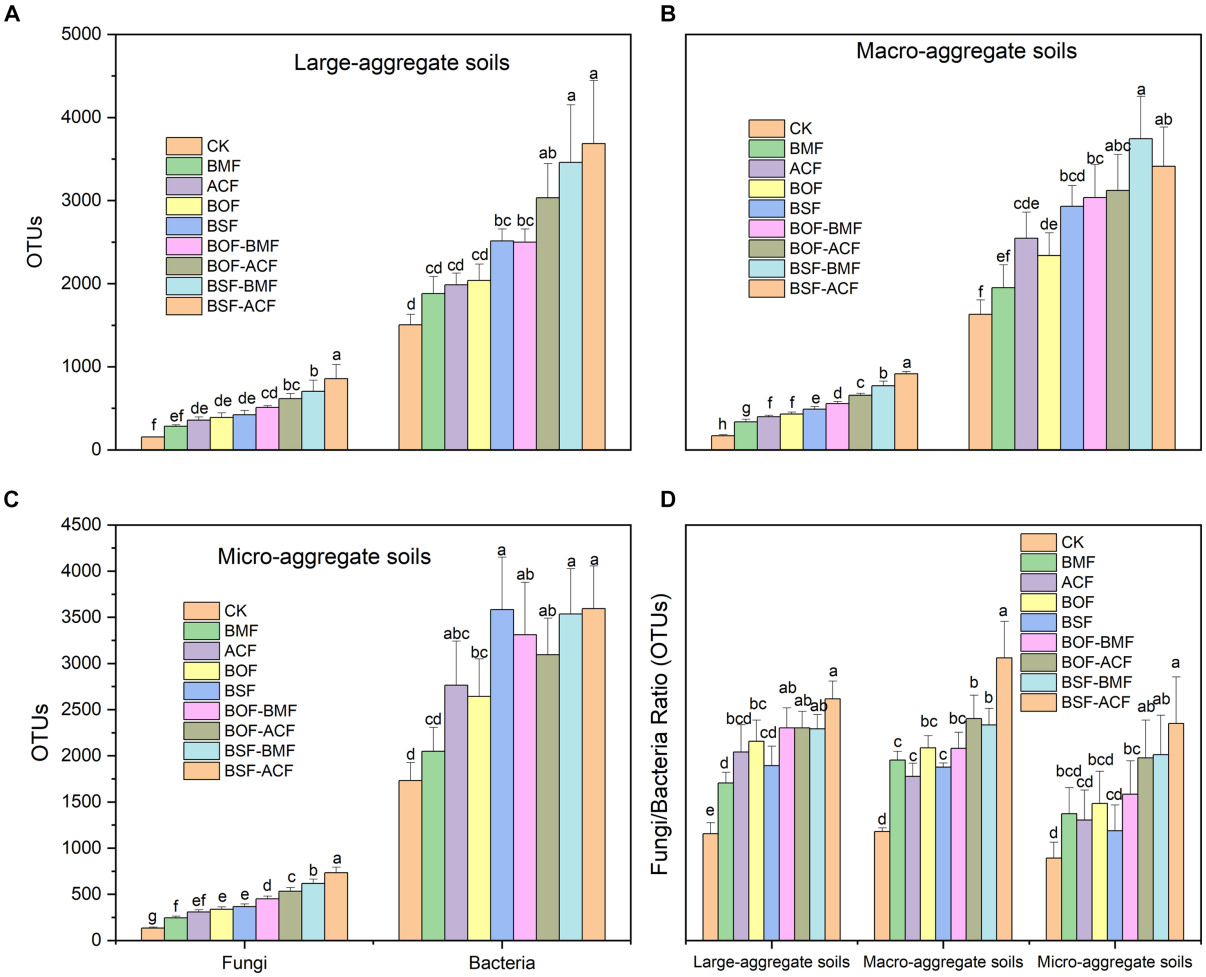
Soil microbial OTU contents under the micro-organic fertilizers in *Chimonobambusa hejiangensis* forest. The values not sharing the same lowercase letters in the bars are significantly different (*p* < 0.05) according to Fisher’s LSD test. The values are means ± SD, *n* = 9. Different uppercase letters indicate significant differences among soil aggregate fractions regardless of fertilizer type (Fisher’s LSD test, *p* < 0.05). CK means no fertilizers. BMF means *Bacillus mucilaginosus* microbial fertilizer. ACF means *Azotobacter chroococcum* microbial fertilizer. BOF means organic fertilizer. BSF means special organic fertilizer for bamboo shoots. BOF + BMF means organic fertilizer plus *Bacillus mucilaginosus*. BOF + ACF means organic fertilizer plus *Azotobacter chroococcum*. BSF + BMF means special organic fertilizer for bamboo shoots plus *Bacillus mucilaginosus*. BSF + ACF means special organic fertilizer for bamboo shoots plus *Azotobacter chroococcum*. Large-aggregate soil (2–4 mm), macro-aggregate soil (0.25–2 mm), and micro-aggregate (<0.25 mm).

In general, soil fungal OUTs, bacterial OUTs, and the ratio of fungal OUTs to bacterial OUTs grew to varied degrees across all aggregates, regardless of whether organic fertilizer or microbial fertilizer was performed alone or in combination (Figures 3A–C). The degree of increase in fungal OUTs following fertilizer is slightly lower for large-aggregate soils compared to macro- and micro-aggregate soils. This suggested that relative smaller soil aggregates were more receptive to fungal colonization when exposed to external fertilization (Figures 3A–C). By combining organic fertilizer with microbial fertilizer, the number of bacterial OUTs increased significantly in different dimensions of soil aggregates (Figures 3A–C). In particular, not all soil aggregates exhibited a statistically significant rise in bacterial OUT numbers when treated with BMF. The mixture of organic fertilizer and microbial fertilizer has a higher capacity to enhance the content of soil bacterial OTUs compared to adopting just microbial fertilizer or organic fertilizer alone (Figures 3A–C). Pre- and post-fertilization, the number of bacterial OTUs in all aggregates was much higher than the number of fungal OUTs. In contrast, fungal OTUs grew more rapidly than bacterial OTUs after fertilization, especially in larger soil aggregates (Figure 3D).

### 3.4 Linkages between microbial necromass traits and soil living microbial and environmental variables

**Figure 4:**
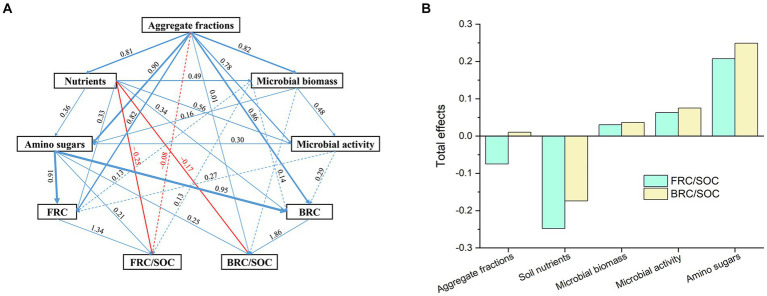
Partial least squares path modeling for the effects of soil aggregate fractions **(A)** and nutrients microbial necromass carbon and their contribution to soil organic carbon **(B)**. Blue line represent positive paths, red line represent negative paths. The width of lines stands for effect level, the numerical values along the lines stands for the effect level and the broken line represent indirect effects. Soil aggregate fractions were classified as large-aggregate (2–4 mm), macro-aggregate (0.25–2 mm), and micro-aggregate (<0.25 mm) soils. Microbial biomass was represented by microbial biomass carbon, PLFAs, fungal PLFAs, and bacterial PLFAs. Microbial activity was indicated by three soil extracellular enzymes (β-1, 4-glucosidase, β-1, 4-N-acetyl-glucosaminidase, acid phosphatase). Soil amino sugars was indicated by glucosamine (GluN), galactosamine (GlaN), muramate (MurA), and mannosamine (ManN). FRC, fungi residue carbon; BRC, bacterial residue carbon; SOC, soil organic carbon. *n* = 243, goodness of fit = 0.71.

To investigate the networks in mediating the contribution of soil microbial residue attributes under the distinct fertilizer inputs, partial least square path modeling (PLS-PM) was utilized (Figure 4A). Soil nutrient inputs had an adversely influenced the contribution of soil fungal and bacterial microbial residues to SOC, and this nutrient-suppressing effect was primarily attributed to the direct effect (Figure 4B). Soil nutrient addition inclined to a similar increase of microbial biomass and enzyme activity, which was negatively impacted the FRC/SOC in soil (Figure 4A). Moreover, soil aggregate fractions tended to have an indirect negative influence on the contribution of FRC to SOC and an indirect positive impact on the contribution of BRC to SOC (Figure 4B), indicating a larger contribution of FRC to SOC in the smaller aggregate fractions at current fertilizing conditions.

## 4 Discussion

In the present study, regardless of fertilizer types, three years of uninterrupted organic- and micro-organic fertilization increased soil amino sugar concentrations compared with the unfertilized plots. Persistent fertilizer application resulted in the accumulation of organic matter in soils (Table 2), which favoured microbial proliferation (Figure 2) and residue gathering (Figure 2). Previous studies have shown that organic fertilizer addition in farming systems increases the soil microbial residual carbon (Schmidt et al., 2017; Zhao et al., 2019; Samson et al., 2020). These findings also demonstrated that the accumulation of soil microbial residual carbon positively correlated with boom of soil microorganisms (Figure 2). Moreover, organic manure addition usually causes a differential response of soil fungi and bacterial residues, with negative effects for fungal and positive effects for bacterial (Sradnick et al., 2014; Ding et al., 2015; Zhao et al., 2019). The difference, however, is that soil microbial fungal and bacterial detritus exhibited a similar increasing trend in our study (Table 2). The distinct feedbacks of soil fungal and bacterial communities in cropland were highly due to the exogenous labile organic carbon input by nourishing the reproduction of bacterial communities (Li et al., 2020) and the delayed replenishment of recalcitrant organic carbon by biomass harvesting (Xu et al., 2020). While the input of organic fertilizer in our study not only supplied easily available substrates from manure but also enlarged the recalcitrant carbon pool (Wang G. et al., 2016; Ghosh et al., 2018) from bamboo litter. This scenario ultimately led to the living and necromass booming of fungal and bacterial in soil (Figure 2 and Table 2). Additionally, the combination of organic and microbial fertilizer produced more microbial necromass carbon concentration than individual organic fertilizer and controls (Figure 2). The combinational treatments produced more soil microbial biomass (Table 2), which contributed to the accrual of microbial residue carbon (Figure 2). Soil organic nutrient levels did improve marginally after applying microbial fertilizers; however, microbial residue content actually decreased. This is likely because the soil organic nutrients provided by the microbial fertilizer treatments were stored in the soil macroaggregates (Tisdall and Oades, 1982), while the soil microaggregates were unable to take up any more microbial residues, resulting in a net loss of microbial residues. After all, small soil aggregates are the primary source of microbial residue buildup (Luan et al., 2021). Moreover, ACF was more effective on soil microbial living and necromass abundances than BMF either alone or in combination in our study (Table 2 and Figure 3). It is highly possible that the functional differences between ACF and BMF caused distinct yields of soil microbial biomass and necromass. Because ACF is a nitrogen, phosphorus, and phosphorus solubilizing bacterium, whereas BMF is a primarily nitrogen-fixing bacterium (García-Fraile et al., 2015). Even though BMF has a greater ability to generate a soil N-rich scenario (Table 2), ACF appears to provide comprehensive nutrients for microbial growth and microbial residual carbon deposition (Figure 3). Collectively, the previously discussed information indicates that the inherent qualities of various fertilizers have a bearing on the features of soil fertility. Furthermore, when coupled with the properties of soil aggregates, these fertilizer properties eventually influence the retention characteristics of soil microbial residues. These findings also confirmed and aligned with our first hypothesis.

As much as 35% of the SOC in forest topsoil was produced by soil microbial necromass carbon globally (Wang B. et al., 2021; Wang C. et al., 2021). In our investigation, this proportion was closer to 45 percent (Table 2). The formation of edaphons, which is beneficial to microbial residue buildup, may be responsible for the unique litter features of bamboo with a somewhat greater return rate (Luan et al., 2021). Additionally, soil N-rich circumstances in microbial waste preferentially connect to the mineral surfaces, safeguarding the microbial leftover carbon from being consumed by bacteria (Kopittke et al., 2018). Soils with bigger aggregates exhibited a non-significant decrease in MRC/SOC following fertilization, whereas those with smaller aggregates showed a little rise (Figure 4). The results demonstrated that SOC accumulated more efficiently in bigger aggregates compared to microbial residues, whereas the opposite was true for smaller aggregates. Furthermore, the microbial necromass accumulation coefficient (Figure 4) suggests that small aggregates were more appropriate for the production of microbial leftovers that are not consumed by bacteria and are retained due to physical or chemical protection (Kleber et al., 2021). Fertilization also caused a difference in the residual carbon contributions to SOC supplied by fungi and bacteria in the large aggregate and microaggregate fractions (Figure 4). Since fungi prefer macro-aggregates and bacteria prefer micro-aggregates (Kumar et al., 2017; Lehmann et al., 2017; Murugan et al., 2019), the distribution of microbial remnants inside aggregates is generally determined by the microhabitats produced by soil aggregates. Thus, it follows that bacterial growth in microaggregates would increase residue accumulation and raise BRC/SOC, just as fungal colonization of large aggregates would consume soil nutrients and increase residue accumulation, leading to an increase in FRC/SOC. However, after fertilization, we observed the opposite effect (Figure 2). Most likely, the results we observed may be attributed to the use of organic and microbial fertilizers. Microbial fertilizer used in this study was a bacterial one, and its colonization altered the soil’s F:B structure (Table 2) by consuming nutrients, including fungal byproducts. In contrast, organic fertilizer, being composed of readily decomposable carbon, facilitates the proliferation of *r*-strategic bacteria (Yuan et al., 2021), hence enhancing the use of chitin generated from fungi (Sinsabaugh et al., 2008; Schnecker et al., 2019). Furthermore, as posited by the “hunger games” concept (Dai et al., 2022), environments abundant in nutrients tend to promote the proliferation of bacteria with rapid growth rates, whereas resource-limited conditions favor the survival of bacteria that exhibit effective nutrient use strategies. This might explain why there seems to be an increasing amount of bacterial necromass in the soil now. It seems that the presence of organic matter in bamboo forests creates an unfavorable environment for the establishment of slow-growing fungi, which are classified as *k*-strategists (Yuan et al., 2021). The contribution of fungal or bacterial residues to soil organic carbon (SOC) is often determined by the equilibrium between microbial product production and breakdown (Joergensen, 2018), as well as the nature of soil nutrient provision (Yuan et al., 2021).

According to the findings of Yuan et al. (2021), fertilizer application (inorganic fertilizer) has a limited or even negative effect on microbial residues in soil aggregates. Our study also revealed a detrimental impact of microbial residue accumulation following microbial fertilizer application (Figure 2). These results highlighted the sensitivity and dynamic features of soil aggregates, especially the microaggregate variability. Similarly, when combining microbial and organic fertilizers, microaggregates demonstrated higher sensitivity to changes in microbial residues than large aggregates (Figure 2). This research also found that fungal remnants differ considerably between big and tiny aggregates, with the latter displaying more striking differences. This demonstrated that fungal residues tend to assemble at a smaller scale than macroaggregates. This not just went against our second hypothesis, but also contradicts the findings of previous research (Spurgeon et al., 2013; Murugan et al., 2019; Yuan et al., 2021). To account for the turnover of tiny aggregates, we hypothesized that the more common fungal residues would serve as additional aggregation sites for new micro-aggregates (Upton et al., 2019). Our findings highlighted that soil aggregates exert considerable influence on the distribution of soil microbial residues, but that the interactions between aggregate components and exogenous nutrients appear to be extremely complex, suggesting that soil aggregates play only a weak protective role in regulating microbial residues with respect to nutrient availability. The balance of degradation and production of their necromass was mediated by various biotic and abiotic residue factors, such as nutrient availability, microbial interaction, physical protection (Powers et al., 2015), aggregate turnover (Six et al., 2000), and exchange of aggregate fraction (Wilpiszeski et al., 2019).

## 5 Conclusion

Altogether, we found that adding either microorganisms or organic fertilizer alone did not cause statistically significant changes in the amount of carbon left over by microorganisms in different soil aggregates, but that using both together made a significant difference. This pattern was seen for several soil properties, including microbial biomass (PLFAs), microbial OUTs, enzyme activity, and total organic carbon and nitrogen in soil aggregates. These variations illustrated the turnover rate and sensitivity of soil aggregates under various fertilizing regimes. *Azotobacter chroococcum* fertilizer has a better chance of keeping microbial residues than *Bacillus mucilaginosus* fertilizer, whether used alone or together. In this experiment, fertilizing behavior caused a slight decrease in MRC/SOC, which a reduction in soil aggregate size largely offset. PLS-PM found that in micro-aggregate soils, soil fungal residues contributed an increasing fraction of SOC while soil bacterial residues contributed a decreasing fraction. These results revealed that fungal residues are the primary binding material or focal point for the formation of new small aggregates at this stage. Our results showed that the ways in which bacteria and fungi respond to the addition of micro- or organic-fertilizers in this bamboo forest soils are probably different. This information is critical for accurately modeling soil microbial residue variations and anticipating C-cycle feedback in *Chimonobambusa hejiangensis* forests.

## Data availability statement

The raw data supporting the conclusions of this article will be made available by the authors, without undue reservation.

## Ethics statement

The manuscript presents research on animals that do not require ethical approval for their study.

## Author contributions

XC: Writing – original draft, Visualization, Methodology, Investigation, Data curation. ZL: Writing – review & editing, Writing – original draft, Visualization, Software, Formal analysis, Data curation. YL: Writing – original draft, Visualization, Investigation, Data curation. YC: Writing – review & editing, Validation, Supervision, Conceptualization. BW: Writing – original draft, Visualization, Investigation, Conceptualization. BZ: Writing – review & editing, Validation, Supervision, Data curation. PR: Writing – review & editing, Validation, Software, Conceptualization. XZ: Writing – review & editing, Validation, Conceptualization. YH: Writing – review & editing, Validation, Data curation. XL: Writing – review & editing. SH: Writing – review & editing, Supervision, Conceptualization. GX: Writing – review & editing, Supervision, Funding acquisition, Conceptualization.

## Footnotes

^1^www.majorbio.com
